# Luteolin in Safflower Leaves Suppresses Microglial Inflammation Through FOXO3-Mediated *Trem2* Transcription

**DOI:** 10.3390/antiox14121495

**Published:** 2025-12-12

**Authors:** Tiantian Zhang, Shuangxi Zhang, Jiayang Ma, Dmitrii Atiakshin, Shujun Han, Mami Noda, Midori Hiramatsu, Jiankang Liu, Yunhua Peng, Jiangang Long

**Affiliations:** 1 Center for Mitochondrial Biology and Medicine, The Key Laboratory of Biomedical Information Engineering of Ministry of Education, School of Life Science and Technology, Xi’an Jiaotong University, 28 West Xianning Road, Xi’an 710049, China; 2 Research and Educational Resource Center for Immunophenotyping, Digital Spatial Profiling and Ultrastructural Analysis Innovative Technologies, Peoples’ Friendship University of Russia, 6 Miklukho-Maklaya St., Moscow 117198, Russia; 3 Faculty of Public Interest, Tohoku University of Community Service and Science, 3-5-1 Iimoriyama, Sakata 998-8580, Yamagata, Japan; 4 School of Health and Life Science, University of Health and Rehabilitation Sciences, Qingdao 266071, China

**Keywords:** safflower leaves, Alzheimer’s disease, microglia, luteolin, triggering receptor expressed on myeloid cells 2

## Abstract

Neuroinflammation driven by microglial activation is a hallmark of Alzheimer’s disease (AD). Triggering receptor expressed on myeloid cells 2 (TREM2) is a key regulator of microglial inflammation, yet strategies to modulate its expression remain limited. Safflower leaves, a vegetable rich in flavonoids—particularly luteolin—were previously shown to attenuate neuroinflammation, reduce oxidative stress, and ameliorate cognitive impairment in APP/PS1 mice. Here, we demonstrated that safflower leaves inhibit microglial inflammation and upregulate TREM2 in APP/PS1 mice. Luteolin, the major active flavonoid in safflower leaves, exerted anti-inflammatory effects in lipopolysaccharides (LPS)-activated microglia. Mechanistically, luteolin enhanced *Trem2* transcription by activating forkhead box protein O3 (FOXO3), a novel transcriptional regulator of *Trem2* identified through promoter analysis. FOXO3 binding to the *Trem2* promoter was essential for this regulation, and luteolin further promoted FOXO3 nuclear translocation. Crucially, *Trem2* knockdown attenuated luteolin’s anti-inflammatory effects, confirming TREM2 as a key mediator. Overall, our study reveals the FOXO3-TREM2 axis as a potential therapeutic target for neuroinflammation and highlights luteolin present in safflower leaves as a candidate dietary intervention for AD, providing new mechanistic insights into the anti-inflammatory activity of this natural antioxidant.

## 1. Introduction

According to the China Alzheimer Report 2024, Alzheimer’s disease (AD) has become the fifth leading cause of death among both urban and rural residents in China [1]. AD is characterized by progressive neuroinflammation, in which microglia play a pivotal role [2,3,4]. Accumulating evidence strengthens the case for the infectious hypothesis of AD, and viral or bacterial pathogens have been shown to trigger dysregulation of cytokines and brain damage through various pathways, such as microglial activation [5,6]. Under pathological conditions, microglial activation promotes the release of pro-inflammatory mediators such as tumor necrosis factor-α (TNF-α), interleukin-1β (IL-1β), and nitric oxide (NO), thereby contributing to neuronal damage [7,8,9,10]. In AD, several molecules associated with microglial inflammation have been implicated, including triggering receptor expressed on myeloid cells 2 (TREM2), cluster of differentiation 33 (CD33), ATP-binding cassette sub-family A member 7 (ABCA7), Src homology 2 domain-containing inositol phosphatase 1 (SHIP1), and apolipoprotein E (APOE) [11]. TREM2, a transmembrane receptor of the immunoglobulin superfamily, is expressed predominantly by microglia in the central nervous system (CNS) [12], and engages in multiple molecular interactions.

Some dietary components with anti-inflammatory and antioxidant properties, such as flavonoids, have beneficial effects on neuroinflammation [13]. Safflower (*Carthamus tinctorius* L.) leaves are regarded in Ayurveda as stomachic, diuretic, and laxative [14], and are consumed as a vegetable in several countries, including Japan and Myanmar [15,16]. Moreover, safflower leaves have been incorporated into a variety of patented functional tea formulations [17,18,19]. Studies have shown that safflower leaves can serve as a dietary immune modulator in poultry [20,21], exhibit considerable 1,1-diphenyl-2-picrylhydrazyl (DPPH) radical scavenging activity [22], and that their methanol extract protects the liver in rats and displays cytotoxicity against human hepatoma cells [14]. Safflower leaves exhibit anti-inflammatory, antioxidant, and anticancer effects [23]. Safflower leaves are rich in flavonoids, such as luteolin and quercetin [22]. Luteolin was first isolated from mignonette and is widely present in vegetables, fruits, and medicinal herbs; it exerts antioxidant, anti-inflammatory, cardioprotective, neuroprotective, and anti-allergic activities [24,25].

Building on the antioxidant and anti-inflammatory activities of safflower leaves, our previous study demonstrated that they can ameliorate cognitive impairment, decrease inflammatory factors, reduce lipid peroxidation products and suppress excessive astrocyte activation in APP/PS1 mice [26]. However, whether safflower leaves regulate microglial inflammation and what the underlying mechanisms remain unknown. Therefore, in this study, we investigated whether safflower leaves inhibit microglial inflammation in APP/PS1 mice and identified the active flavonoids responsible, in order to further elucidate the mechanisms underlying its anti-inflammatory effects.

## 2. Materials and Methods

### 2.1. Animals

This study utilized the B6C3-Tg (APPswePSEN1dE9) mouse model, one of the most widely used and well-characterized models of AD amyloid pathology. This transgenic line is hereafter referred to as APP/PS1. The feeding and treatments of mice were described previously [26]. A total of 45 three-month-old female APP/PS1 mice and age-matched wild-type (WT) littermates were purchased from the Nanjing Biomedical Research Institute of Nanjing University, China. Mice were housed under specific pathogen free conditions in a controlled environment (23–25 °C, 60% humidity, 12 h light/dark cycle) with free access to food and water. Mice were randomly divided into three groups (n = 15 per group): WT + H_2_O, APP/PS1 + H_2_O, and APP/PS1 + safflower leaves powder (SLP). Mice in the APP/PS1 + SLP group received SLP (100 mg/kg/day) dissolved in water by gavage, whereas the WT + H_2_O and APP/PS1 + H_2_O groups received water only. The sample size was chosen based on common practices in long-term studies of AD models to account for potential variability and attrition. All procedures involving animals were approved by the Biomedical Ethics Committee of Health Science Center of Xi’an Jiaotong University (XJTUAE2025-3716). After 10 months of treatment and subsequent behavioral testing, all mice were sacrificed, and the left hemispheres were fixed in 4% paraformaldehyde (PFA) for staining. Investigators were blinded to the group allocation during outcome assessment.

### 2.2. Cell Culture

N9 cells (ATCC, Manassas, VA, USA) were cultured in RPMI-1640 medium with 10% fetal bovine serum (FBS) (NEWZERUM, Christchurch, New Zealand). RAW264.7 and 3T3-L1 cells were cultured in high glucose-Dulbecco’s modified eagle medium (H-DMEM) with 10% FBS. All cells were incubated at 37 °C incubator containing 5% CO_2_ and were passaged every three days. After cells were seeded for 24 h, RAW264.7 cells were pretreated with different concentrations of luteolin (Aladdin, Shanghai, China) or quercetin (Aladdin, Shanghai, China) for 2 h, and then treated with 0.1 μg/mL lipopolysaccharides (LPS) (Sigma-Aldrich, Darmstadt, Germany) for 3 h. Determination of optimal LPS treatment conditions was established through preliminary optimization experiments (Supplementary Figure S1). After cells were seeded for 24 h, N9 cells were pretreated with 40 μM luteolin for 2 h, and then treated with 1 μg/mL LPS for 10 h. Cells were then harvested for staining, and protein or RNA extraction.

### 2.3. Cell Viability

Cell viability was assessed using 3-(4,5-dimethylthiazol-2-yl)-2,5-diphenyltetrazolium bromide (MTT). After adding MTT to the wells, cells were incubated at 37 °C for 4 h. Subsequently, the supernatant in the 96-well plates was removed, and 150 μL of dimethyl sulfoxide (DMSO) was added to each well. Finally, the absorbance at 495 nm was measured using a microplate reader.

### 2.4. Calcein AM Staining

N9 cells in 12-well plates were washed with PBS, after which 500 μL of 0.5 μM Calcein AM was added. Following a 30-min incubation at 37 °C in the dark, the Calcein AM solution was removed, and fresh culture medium was added for a further 30-min incubation at 37 °C. Finally, the cells were washed twice with PBS and observed under the microscope.

### 2.5. Immunofluorescence (IF)

For staining of the mouse brain slices, IF was performed at Servicebio Technology Co., Ltd., Wuhan, China, as described previously [26]. For cells IF staining, cell slides were placed into new six-well plates and washed twice with phosphate-buffered saline (PBS). Slides were then fixed with 4% PFA for 15 min and washed with PBS. The slides were then permeabilized with 0.5% Triton X-100 in PBS for 20 min at room temperature. After three washes, the slides were blocked with 3% bovine serum albumin (BSA) in PBS for 30 min. The slides were then incubated with primary antibodies for 2 h at room temperature, washed three times with PBS, incubated with secondary antibodies for 2 h, washed three times, stained with 4′,6-diamidino-2-phenylindole (DAPI) for 5 min, and washed three times at room temperature. Finally, the slices were mounted with 50% glycerin. The cluster of differentiation 68 (CD68) antibody (Abclonal, Wuhan, China), diluted 1:100 in PBS containing 1% BSA, were used to stain activated microglia.

### 2.6. Multiplex Immunohistochemistry (mIHC)

Sequential mIHC for the simultaneous detection of ionized calcium-binding adaptor molecule 1 (Iba1), forkhead box protein O3 (FOXO3) and TREM2 in mouse brains was performed in accordance with Akoya Biosciences (Marlborough, MA, USA) recommendations on the use of OPAL series fluorochromes for the Mantra 2 Quantitative Pathology Imaging System (OPAL 480, 540 and 650). In addition, when using OPAL series fluorochromes (Akoya Biosciences, Marlborough, MA, USA) for repeated retrieval, the EZ-Retriever System, MW015-IR (BioGenex, Fremont, CA, USA) was applied. Stained sections were observed using a ZEISS Axio Imager.Z2 (Carl Zeiss Vision, Jena, Germany).

### 2.7. Western Blot

Cells were lysed by cell lysis buffer (Beyotime, Shanghai, China) supplemented with phenylmethylsulfonyl fluoride (PMSF). Lysates were centrifuged at 13,000 rpm for 15 min at 4 °C, and the supernatants were collected. Protein concentrations were measured using the Pierce™ BCA Protein Assay Kit (Thermo Fisher Scientific, Rockford, IL, USA). Western blot was performed as described previously [26]. The TREM2 antibody (Affinity, Zhenjiang, China), spleen tyrosine kinase (SYK) antibody (Abclonal, Wuhan, China), p-SYK antibody (CST, Danvers, MA, USA), toll-like receptor 4 (TLR4) (Santa Cruz Biotechnology, Dallas, TX, USA), FOXO3 antibody (CST, Danvers, MA, USA), extracellular signal-regulated kinase (ERK) antibody (CST, Danvers, MA, USA) and p-ERK antibody (CST, Danvers, MA, USA) were diluted 1:1000 in 1% BSA prepared in Tris-buffered saline with Tween 20 (TBST). The β-ACTIN antibody (CST, Danvers, MA, USA), GAPDH antibody (CST, Danvers, MA, USA) and TUBULIN antibody (CST, Danvers, MA, USA) were diluted 1:3000 in 1% BSA prepared in TBST.

### 2.8. Real-Time PCR

Total cellular RNA was extracted using RNAex Pro Reagent (AG, Changsha, China) and reverse-transcribed in cDNA using Evo M-MLV RT Premix (AG, Changsha, China) according to the instructions. And real-time PCR was performed by SYBR^®^ Green Premix *Pro Taq* HS qPCR Kit (AG, Changsha, China). The primers used are listed in Supplementary Table S1.

### 2.9. Transfection

For siRNA transfection, cells in 6-well plates at approximately 40% confluence were transfected using Lipofectamine™ RNAiMAX (Invitrogen, Carlsbad, CA, USA) with 25 nM siRNA. And for plasmid transfection, cells in 12-well plates at approximately 50% confluence were transfected using Lipofectamine™ 2000 (Invitrogen, Carlsbad, CA, USA) with the indicated amounts of plasmid for each specific experiment. Both procedures performed according to the instructions. Cells were harvested for analysis 48 h after transfection. The *Trem2* and *Foxo3* siRNA used was purchased from GenePharma Co., Ltd., Shanghai, China, and the sequence is shown in Supplementary Table S2. And the FOXO3 overexpression plasmid used were purchased from MiaoLingBio Co., Ltd., Wuhan, China.

### 2.10. Dual-Luciferase Reporter Assay

The mouse *Trem2* promoter sequence was inserted into pGL3.0-basic plasmid to construct *Trem2* promoter plasmid. Using this plasmid as the template, the *Trem2* promoter mutant plasmid was generated by site-directed mutagenesis PCR (primer sequences in Supplementary Table S3). The *Trem2* promoter plasmid or its mutant variant (1.0 μg) was co-transfected with the pRL-TK plasmid (25.0 ng) into 3T3-L1 cells seeded in 12-well plates. Subsequently, the dual-luciferase reporter assay was performed using the Dual Luciferase Reporter Gene Assay Kit (Beyotime, Shanghai, China) according to the instructions. Firefly luciferase activity was normalized to Renilla luciferase activity to obtain the relative luciferase activity.

### 2.11. Statistical Analysis

All data are presented as the mean ± standard error of the mean (SEM). All statistical analyses were performed using GraphPad Prism software, version 9.0. One-way analysis of variance (ANOVA) was used for comparisons among three or more groups, and Student’s *t* test was used for comparisons between two groups. *p* < 0.05 was considered statistically significant.

## 3. Results

### 3.1. Safflower Leaves Inhibited Microglial Activation in APP/PS1 Mice

**Figure 1:**
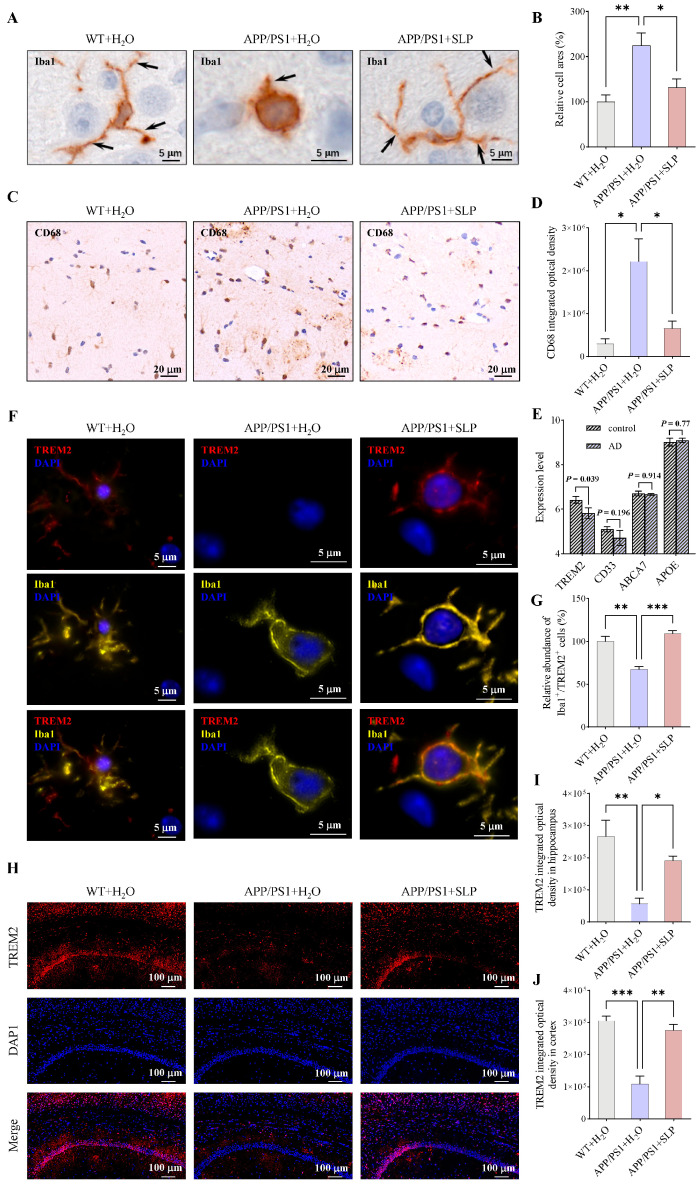
Safflower leaves inhibited microglial activation in APP/PS1 mice. (**A**) Ionized calcium-binding adaptor molecule 1 (Iba1) immunohistochemistry (IHC) in the brain. The arrow points to a microglial process. (**B**) Statistical analysis of Iba1 positive microglia area, n = 3 per group. (**C**) Cluster of differentiation 68 (CD68) IHC in the brain. (**D**) Statistical analysis of CD68 integrated optical density, n = 3 per group. (**E**) Expression of microglial inflammatory genes in a public Alzheimer’s disease (AD) dataset. Data was retrieved directly from the AlzData database (visualization of GEO dataset GSE26972), n = 3 per group. (**F**) Iba1 and triggering receptor expressed on myeloid cells 2 (TREM2) colocalization. (**G**) Relative abundance of Iba1^+^/TREM2^+^ cells in the brain, n = 3 per group. (**H**) TREM2 immunofluorescence (IF) in the brain. (**I**,**J**) Statistical analysis of TREM2 integrated optical density in the hippocampus (**I**) and the cortex (**J**), n = 3 per group. Values represent the mean ± SEM. The results were analyzed by one-way ANOVA. * *p* < 0.05, ** *p* < 0.01, *** *p* < 0.001.

Microglia activation is accompanied by morphological changes and CD68 up-regulation [27]. To investigate whether safflower leaves can inhibit microglial inflammation in APP/PS1 mice, microglial markers Iba1 and CD68 were detected in the brain by IHC. For Iba1 staining, compared with the WT + H_2_O group, the APP/PS1 + H_2_O group exhibited enlarged cell area and shortened processes, whereas SLP treatment ameliorated these morphological alterations in microglia (Figure 1A,B). And the 3D model of microglia in the brain was presented in a video format (Supplementary Figure S2). For CD68, integrated optical density was significantly increased in APP/PS1 + H_2_O mice compared with WT + H_2_O mice (Figure 1C,D). SLP treatment significantly decreased CD68 integrated optical density in APP/PS1 + SLP mice (Figure 1C,D). Consistent with our hypothesis, safflower leaves attenuated microglial activation in APP/PS1 mice.

Using the AlzData database [28], we analyzed the expression levels of microglial inflammatory genes in AD patients based on the GEO dataset GSE26972. It was found that *TREM2* expression was reduced in AD patients (Figure 1E). Given the crucial role of TREM2 in regulating microglial inflammation, we subsequently measured the TREM2 level in APP/PS1 mice by IF. In CNS, TREM2 is expressed in microglia [12]. Through co-staining of Iba1 and TREM2, we also found that TREM2 is expressed in microglia (Figure 1F). The proportion of TREM2-positive microglia was reduced in the APP/PS1 + H_2_O group compared to the WT + H_2_O group, and this reduction was reversed by safflower leaves treatment (Figure 1G). Mice in the APP/PS1 + H_2_O group showed lower TREM2 immunofluorescence intensity both in the hippocampus and cortex than WT + H_2_O mice (Figure 1H–J), and SLP treatment increased the immunofluorescence intensity of TREM2 in APP/PS1 + SLP group mice (Figure 1H–J). These results suggest that TREM2 may be a key mediator of safflower leaf–mediated modulation of microglial inflammation.

### 3.2. Luteolin Served as the Major Active Flavonoid in Safflower Leaves

Multiple flavonoids in safflower leaves have been shown to have anti-AD activity [29]. To elucidate how safflower leaves suppressed microglial inflammation in APP/PS1 mice, we selected flavonoids with high content and strong anti-inflammatory effect in safflower leaves as the major effective flavonoid. We previously found that, among the six flavonoids analyzed ((±)-catechin hydrate, (−)-epicatechin gallate, cynaroside, quercetin, luteolin, and (−)-gallocatechin) in safflower leaves, the contents of cynaroside, luteolin and quercetin were significantly higher than those of the others [26]. Considering that cynaroside is hydrolyzed to luteolin prior to absorption [30], we compared the anti-inflammatory effects of luteolin and quercetin in LPS-activated RAW264.7 cells.

**Figure 2:**
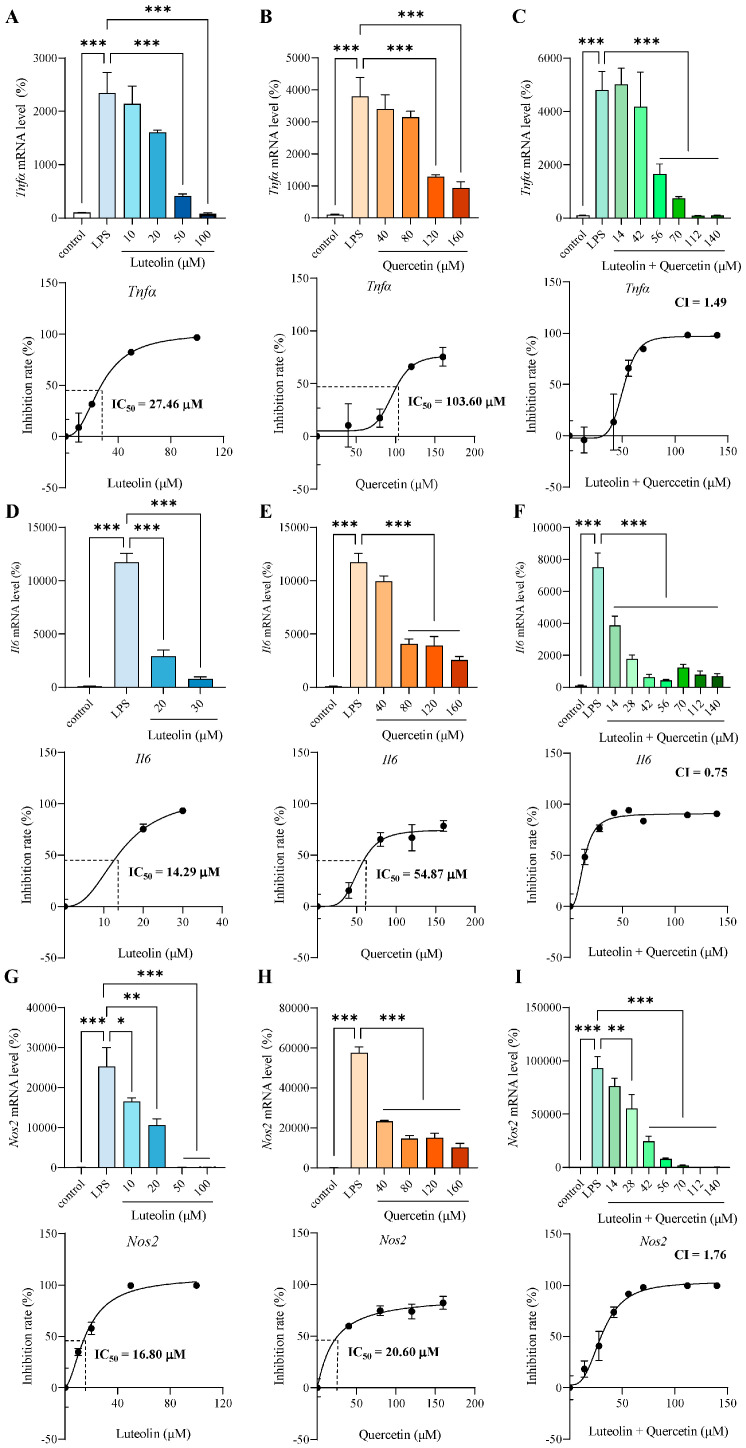
Luteolin served as the major active flavonoid in safflower leaves. (**A**–**C**) mRNA level and inhibition rate of tumor necrosis factor α (*Tnfα*) pretreated for 2 h with different concentrations of luteolin (**A**), quercetin (**B**), or luteolin + quercetin (mixed at ratios reflecting their relative abundances in safflower leaves) (**C**), followed by 3 h lipopolysaccharides (LPS) stimulation. (**D**–**F**) mRNA level and inhibition rate of interleukin-6 (*Il6*) pretreated for 2 h with different concentrations of luteolin (**D**), quercetin (**E**), or luteolin + quercetin (mixed at ratios reflecting their relative abundances in safflower leaves) (**F**), followed by 3 h LPS stimulation. (**G**–**I**) mRNA level and inhibition rate of nitric oxide synthase 2 (*Nos2*) pretreated for 2 h with different concentrations of luteolin (**G**), quercetin (**H**), or luteolin + quercetin (mixed at ratios reflecting their relative abundances in safflower leaves) (**I**), followed by 3 h LPS stimulation. Half maximal inhibitory concentration (IC_50_) or combination index (CI) values are shown. Values represent the mean ± SEM, n = 3 per group. The results were analyzed by one-way ANOVA. * *p* < 0.05, ** *p* < 0.01, *** *p* < 0.001.

Both luteolin and quercetin dose-dependently inhibited LPS-induced inflammatory mediators, including *Tnfα* (Figure 2A,B), *interleukin-6* (*Il6*) (Figure 2D,E) and *Nos2* (Figure 2G,H). We used GraphPad Prism software to fit the inhibition curve and calculated the half maximal inhibitory concentration (IC_50_). Additionally, cells were treated with mixtures of luteolin and quercetin at various concentrations (mixed at ratios reflecting their relative abundances in safflower leaves) (Figure 2C,F,I). The combination index (CI) was calculated based on the IC_50_ to assess whether luteolin and quercetin exerted synergistic anti-inflammatory effects. Since CI less than 1 indicates synergy, they exhibited a synergistic effect only for *Il6* (Figure 2F). The results showed that for these three inflammatory mediators, the two compounds did not synergize well (Figure 2C,F,I). Therefore, we compared the anti-inflammatory effects of using luteolin or quercetin alone. For the three inflammatory mediators measured, the IC_50_ values of luteolin were consistently lower than those of quercetin, indicating that luteolin has stronger anti-inflammatory ability than quercetin (Figure 2A,B,D,E,G,H). So, we chose luteolin as the major active flavonoid in safflower leaves to explore the molecular mechanism.

### 3.3. Luteolin Inhibited Microglial Inflammatory Response and Increased TREM2 in LPS-Activated N9 Cells

**Figure 3:**
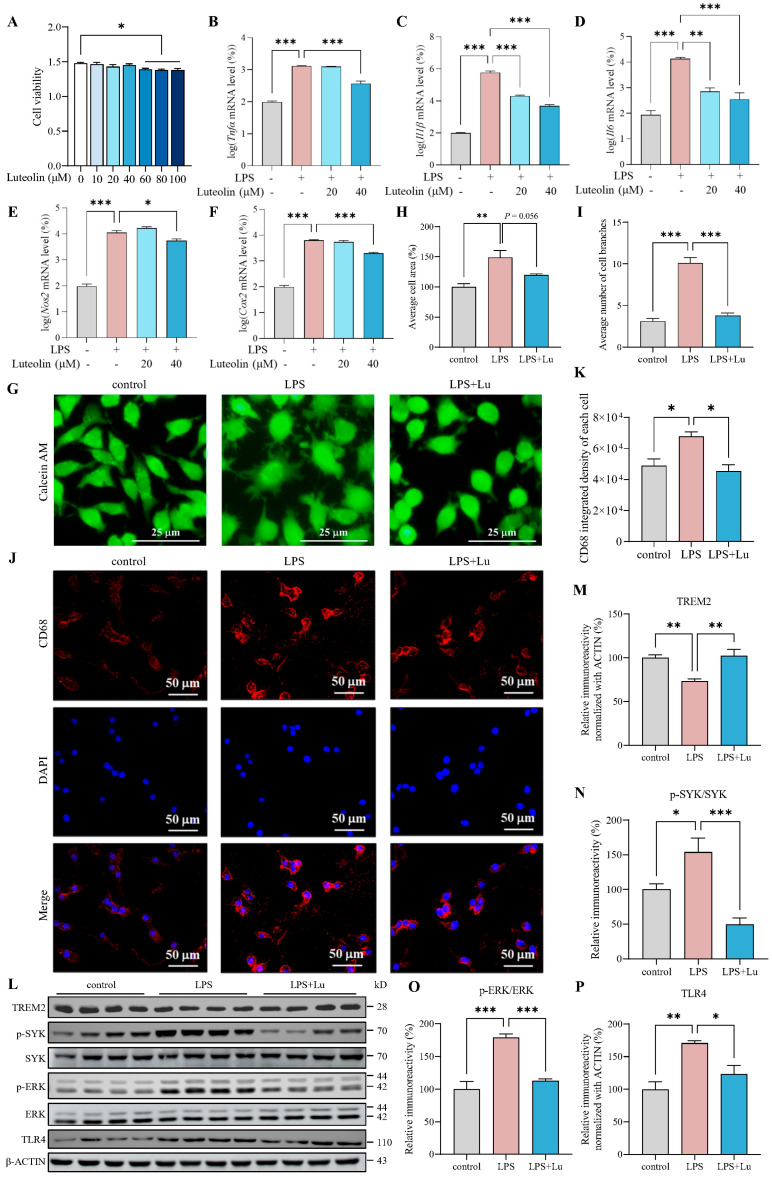
Luteolin inhibited microglial inflammatory responses and increased triggering receptor expressed on myeloid cells 2 (TREM2) in lipopolysaccharides (LPS)-activated N9 cells. (**A**) Cell viability, n = 6 per group. (**B**–**F**) mRNA levels of tumor necrosis factor-α (*Tnfα*), interleukin-1β (*Il1β*), interleukin-6 (*Il6*), nitric oxide synthase 2 (*Nos2*) and cyclooxygenase-2 (*Cox2*), n = 3 per group. (**G**) Calcein AM staining. (**H**,**I**) Statistical analysis of cell area (**H**) and cell branches number (**I**), n = 3 per group. (**J**) Cluster of differentiation 68 (CD68) immunofluorescence (IF). (**K**) Statistical analysis of CD68 integrated density, n = 3 per group. (**L**) Representative images of western blot results for TREM2, p-spleen tyrosine kinase (SYK), SYK, p-extracellular signal-regulated kinase (ERK), ERK, toll-like receptor 4 (TLR4) and β-ACTIN. (**M**–**P**) The results were quantified and normalized to β-ACTIN, n = 4 per group. Values represent the mean ± SEM. The results were analyzed by one-way ANOVA. * *p* < 0.05, ** *p* < 0.01, *** *p* < 0.001.

To determine appropriate luteolin concentrations for N9 cell treatment, cell viability was assessed using MTT after exposure to various concentrations of luteolin. It showed that luteolin concentrations below 40 μM had no significant effect on N9 cell viability (Figure 3A). Based on this result, we used 20 μM and 40 μM luteolin to examine effects on inflammatory mediators such as TNF-α, IL-1β, IL-6, nitric oxide synthase 2 (NOS2) and cyclooxygenase-2 (COX-2) in LPS-activated N9 cells [31,32]. It was found that LPS treatment increased the *Tnfα*, *Il1β*, *Il6*, *Nos2* and *Cox2* mRNA levels, and luteolin ameliorated these LPS-induced elevations, especially 40 μM luteolin can reduce all mRNA levels (Figure 3B–F). Thus, we chose 40 μM luteolin for subsequent experiments with N9 cells.

Acute LPS stimulation induces distinct morphological changes in microglia, characterized by soma enlargement and an increase in shortened processes. This transitional phenotype is considered to mark a shift toward a pro-inflammatory, disease-associated activation state [33,34,35,36]. To investigate the effect of luteolin on the activation of N9 cells, we observed cell morphology through Calcein AM staining and analyzed cell area and number of cell branches using ImageJ software version 1.54f. The results showed that LPS significantly increased the area and branching number of N9 cells, while luteolin partially restored the average cell area and significantly reduced the branching number (Figure 3G–I), indicating that luteolin inhibited the morphological changes of N9 cells induced by LPS. And we detected CD68 by IF. The results showed that LPS significantly increased the average fluorescence density of CD68 in N9 cells (Figure 3J,K). In contrast, luteolin significantly reduced the average fluorescence density of CD68 level (Figure 3J,K). These findings suggested that luteolin can inhibit LPS-induced activation of N9 cells.

When microglia are activated by LPS or inflammatory factors, the expression of TREM2 rapidly decreases [37]. To assess the effect of luteolin on TREM2, we pretreated LPS-induced N9 cells with luteolin, and detected the level of TREM2 by western blot. Compared with control group, the protein level of TREM2 were reduced significantly in the LPS group (Figure 3L,M). And luteolin treatment significantly increased TREM2 levels compared with those in the LPS group, suggesting its role in TREM2 restoration (Figure 3L,M).

LPS binding to TREM2 triggers the TREM2 signaling pathway, leading to SYK phosphorylation and initiation of downstream signaling, such as the ERK pathway [38]. TREM2 can inhibit the TLR response, and its overexpression can suppress TLR4 activation [39,40,41]. The imbalance between TREM2 and TLR4 exacerbates inflammation in BV2 cells [42]. Therefore, we assessed the level of SYK, p-SYK, ERK, p-ERK and TLR4 by western blot. It was found that compared with control group, the levels of p-SYK/SYK, p-ERK/ERK and TLR4 were increased significantly in LPS group (Figure 3L,N–P). And luteolin can decrease the levels of p-SYK/SYK, p-ERK/ERK and TLR4 compared to LPS group (Figure 3L,N–P). These findings suggested that luteolin modulated TREM2 signaling in LPS-activated N9 cells.

### 3.4. Trem2 Knockdown Attenuated Luteolin’s Anti-Inflammatory Effects

**Figure 4:**
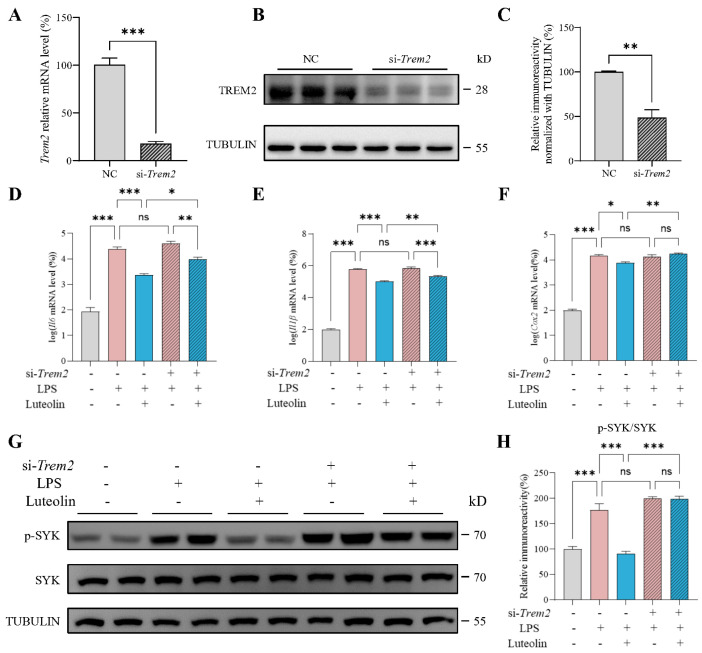
Triggering receptor expressed on myeloid cells 2 (*Trem2*) knockdown attenuated luteolin’s anti-inflammatory effects. (**A**–**C**) mRNA level (**A**) and protein level (**B**,**C**) of TREM2 after knockdown, n = 3 per group. NC represents the negative control. (**D**–**F**) mRNA levels of interleukin-6 (*Il6*), interleukin-1β (*Il1β*) and cyclooxygenase-2 (*Cox2*), n = 4 per group. (**G**) Representative images of western blot results for p-spleen tyrosine kinase (SYK), SYK and TUBULIN. (**H**) Relative immunoreactivity of p-SYK/SYK were quantified, n = 4 per group. Values represent the mean ± SEM. The results were analyzed by one-way ANOVA. * *p* < 0.05, ** *p* < 0.01, *** *p* < 0.001 and ns *p* ≥ 0.05.

To determine whether TREM2 is required for luteolin-mediated inhibition of N9 cell inflammation, we employed siRNA to knock down *Trem2* and examined whether the protective effect of luteolin against LPS-induced inflammation was altered. siRNA effectively reduced the mRNA and protein levels of TREM2, successfully knocking down *Trem2* (Figure 4A–C). Following *Trem2* knockdown, luteolin still reduced the LPS-induced elevation of *Il6* and *Il1β* levels, but the extent of reduction was diminished (Figure 4D,E). Moreover, the reduction on *Cox2* level and the inhibition of SYK phosphorylation by luteolin were abolished after knocking down *Trem2* (Figure 4F–H). These results demonstrated that luteolin’s anti-inflammatory effects were attenuated after *Trem2* knockdown, supporting the crucial role of TREM2 in luteolin-mediated amelioration of inflammation in N9 cells.

### 3.5. Luteolin Promoted Trem2 Transcription Through Increasing FOXO3

**Figure 5:**
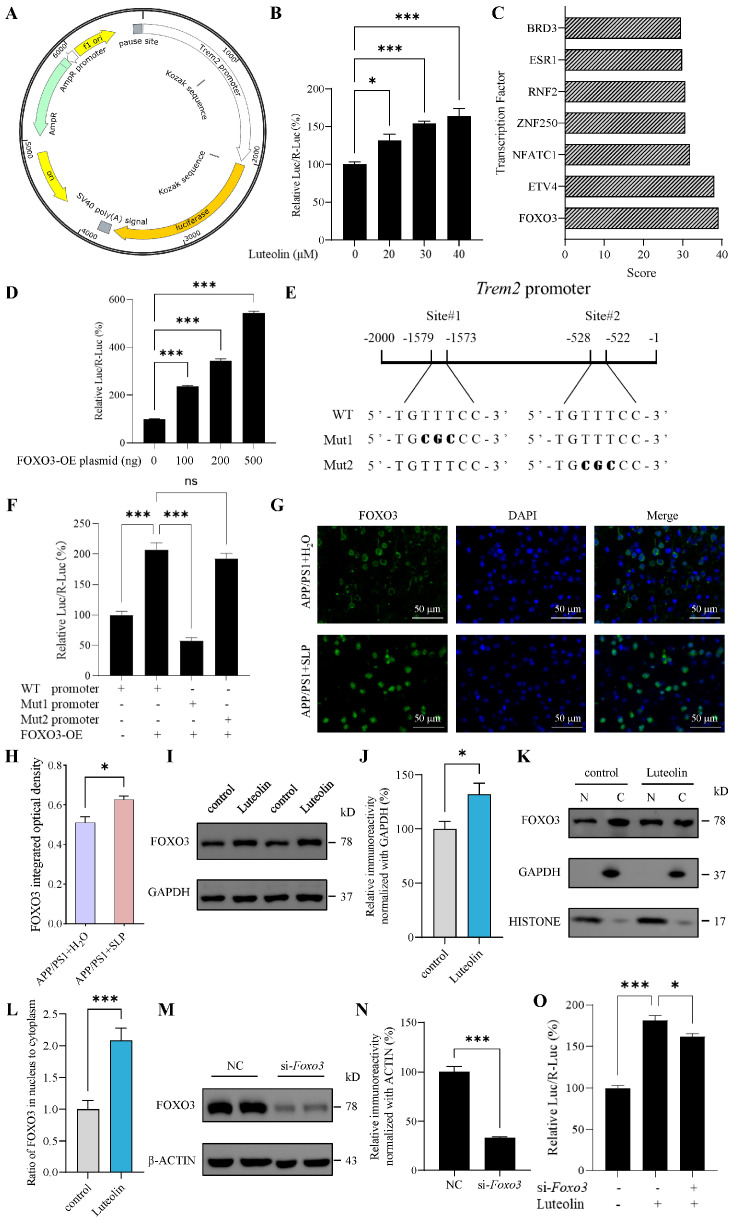
Luteolin promoted triggering receptor expressed on myeloid cells 2 (*Trem2*) transcription by increasing forkhead box protein O3 (FOXO3). (**A**) Plasmid profile of mice *Trem2* promoter constructs in pGL3.0-basic vector. (**B**) *Trem2* promoter activity treated with luteolin in 3T3L1 cells, n = 4 per group. (**C**) Scores for predicting transcription factor binding with *Trem2* promoter using AnimalTFDB3.0 database. (**D**) *Trem2* promoter activity after overexpressing FOXO3 in 3T3L1 cells, n = 4 per group. (**E**) A schematic illustration of *Trem2* promoter region. The positions and sequences of two predicting binding sites of FOXO3 using JASPAR database were marked. The mutated nucleotides of FOXO3 binding sites are shown in bold. (**F**) FOXO3 binding sites mutated-*Trem2* promoter activity after overexpressing FOXO3 (500 ng plasmid) in 3T3L1 cells, n = 6 per group. (**G**) FOXO3 immunofluorescence (IF) in APP/PS1 mice brain. (**H**) Statistical analysis of FOXO3 integrated optical density, n = 3 per group. (**I**) Representative images of western blot results for FOXO3 and β-ACTIN in N9 cells. (**J**) The results were quantified and normalized to β-ACTIN, n = 6 per group. (**K**) Representative images of western blot results for FOXO3, GAPDH and HISTONE in N9 cells. Nuclear (N) and cytoplasmic (C) fractions were isolated. (**L**) Nuclear to cytoplasmic ratio of FOXO3, n = 6 per group. (**M**) Representative images of western blot results for FOXO3 and β-ACTIN after *Foxo3* knockdown in 3T3L1 cells. NC represents the negative control. (**N**) The results were quantified and normalized to β-ACTIN, n = 3 per group. (**O**) *Trem2* promoter activity treated with luteolin and/or siRNA-*Foxo3* in 3T3L1 cells, n = 4 per group. Values represent the mean ± SEM. The results in (**B**–**F**,**O**) were analyzed by one-way ANOVA, the results in (**H**–**N**) were analyzed by Student’s *t* test. * *p* < 0.05, *** *p* < 0.001 and ns *p* ≥ 0.05.

To explore how luteolin regulated TREM2, we first assessed its effect on *Trem2* transcription using dual-luciferase reporter assay. Given the poor transfection efficiency in N9 cells, all plasmid transfection experiments were performed in the readily transfectable 3T3-L1 cells. It was found that luteolin can dose dependently increase the transcriptional activity of *Trem2* promoter (Figure 5A,B). Subsequently, we predicted transcription factors that may bind to mouse *Trem2* promoter using AnimalTFDB3.0 database. FOXO3 ranked highest among the predicted transcription factors (Figure 5C). Therefore, we hypothesized that FOXO3 might act as a transcription factor of *Trem2* and validated this by dual-luciferase reporter assay. It was found that overexpression of FOXO3 can increase the transcriptional activity of *Trem2* promoter, and with the increase of FOXO3 overexpression plasmid, the effect was enhanced (Figure 5D), indicating that FOXO3 is a transcriptional regulator of *Trem2*.

To identify the FOXO3 binding site on the *Trem2* promoter, we introduced point mutations into the predicted binding sites and examined the effect on FOXO3-driven *Trem2* promoter activity. Guided by JASPAR database scores (Supplementary Table S4), we selected the two highest-scoring binding sites for mutation (Figure 5E). It was found that Mut1 abolished the promoting effect of FOXO3 on *Trem2* promoter transcriptional activity, whereas Mut2 did not significantly affect this activity (Figure 5F). Thus, these results identified site#1 as one of the binding sites for FOXO3 on the *Trem2* promoter.

To further investigate whether luteolin regulated *Trem2* transcription via FOXO3, we first examined the effects of luteolin on FOXO3. In APP/PS1 mouse brains, we found that treatment with safflower leaves significantly increased the level of FOXO3 through immunostaining (Figure 5G,H). In N9 cells, it was found that luteolin can increase the level of FOXO3 (Figure 5I,J). To investigate whether luteolin promotes the nuclear translocation of FOXO3, we performed subcellular fractionation. The purity of the fractions was confirmed by using GAPDH as a cytoplasmic control and HISTONE as a nuclear control. It was found that luteolin can increase the ratio of FOXO3 in the nucleus to cytoplasm (Figure 5K,L), which may promote FOXO3 into the nucleus to exert transcriptional regulation. In 3T3-L1 cells, further knockdown of *Foxo3* revealed that the promoting effect of luteolin on *Trem2* promoter transcriptional activity was significantly attenuated (Figure 5M–O), indicating that FOXO3 plays an important role in luteolin-mediated regulation of *Trem2* transcription. Collectively, these results suggested that luteolin promoted *Trem2* transcription, at least in part, by increasing FOXO3.

## 4. Discussion

Flavonoids exhibit multifaceted protection against AD, including amyloid-β (Aβ) clearance, anti-inflammation, antioxidation and cognitive improvement [43,44]. Long-term dietary intake of flavonoids was associated with a lower risk of AD and AD-related dementia in American adults [45]. Consuming certain plants rich in polyphenols or flavonoids can reduce the risk of neuroinflammatory diseases in risk groups [44]. As a flavonoid-rich vegetable, we previously demonstrated that safflower leaves can ameliorate cognitive impairment, reduce oxidative damage, and inhibit neuroinflammation in APP/PS1 mice [26]. Here, we specifically addressed the effects on microglial inflammation, the key driver of AD pathology [8]. Although our previous study did not observe significant effect of safflower leaves on Iba1 levels in APP/PS1 mouse brains, the current research, by comparing the morphology of Iba1-labeled microglia, revealed that safflower leaves restored microglial morphology in APP/PS1 mice, confirming that safflower leaves have an ameliorating effect on microglia-related inflammation in APP/PS1 mice. This finding extends the role of safflower leaves beyond antioxidant activity to include suppression of microglial activation in AD.

Luteolin, a flavonoid known for its anti-inflammatory and antioxidant properties, can inhibit microglial inflammation including suppression of CD40 and reduction of TNF-α and IL-6 [46,47,48]. A co-ultramicronized composite containing luteolin and palmitoylethanolamine has demonstrated neuroprotective effects in stroke patients undergoing neurorehabilitation [49]. Dietary supplements containing luteolin and other bioactive plant compounds may improve cognitive function in AD patients [44]. Our study demonstrated that luteolin suppressed LPS-activated N9 cell inflammation, mirroring the findings on safflower leaves in APP/PS1 mice [26]. Although the luteolin concentration in safflower leaves is markedly lower than the dose reported in studies using luteolin itself in AD mice [50,51], this discrepancy may be attributed to additional bioactive constituents present in safflower leaves. While luteolin and quercetin exhibited limited synergistic anti-inflammatory activity, the possibility of cooperative effects among other, less abundant flavonoids in safflower leaves cannot be excluded. Moreover, the 10-month oral gavage mimics long-term dietary exposure and far exceeds the intervention durations reported for luteolin, while the chronic low-dose administration may drive cumulative effects. LPS, as a stimulator of inflammation, is widely used to activate N9 cells to model the neuroinflammatory aspects of AD for related researched [42,52,53,54]. While it is important to acknowledge that in vitro microglial models of AD have limitations—including limited clinical relevance and an inability to fully replicate the complex physiological changes in vivo—they remain indispensable tools for investigating molecular mechanisms and screening potential therapeutic compounds in AD research and drug development [55].

In the pathogenesis of AD, the relationship between TREM2 and microglial behavior is vital [56]. In the brains of AD patients, reactive microglia are found clustered around Aβ plaques [8]. TREM2 acts as a negative regulator of microglial inflammatory responses [57]. Variants in *TREM2* are closely associated with an increased risk of AD. The most common *R47H TREM2* variant elevates the risk of developing AD by 2~3 times [58]. The overexpression of TREM2 can inhibit the activation of TLR4 and its downstream pro-inflammatory signaling pathways [40]. Furthermore, TREM2 has been shown to suppress LPS-activated microglial responses by inhibiting c-Jun N-terminal kinase (JNK) [59]. The restoration of TREM2 by luteolin aligns with prior studies on flavonoids like anthocyanin, curcumin, resveratrol, and baicalin [24,60,61], but our work further identified FOXO3 as its upstream regulator. FOXO3 can act as a transcription factor that regulates target gene expression and plays a key role in inflammation, apoptosis and oxidative stress [62,63]. In mouse brains, FOXO3 expression declines with advancing age [64]. FOXO3 exerts a protective effect against Aβ, and Aβ oligomers can reduce FOXO3 levels in mouse microglia [65]. In AD models, FOXO3 deficiency has been found to exacerbate Aβ pathology [64]. In this study, we found that safflower leaf treatment and its active flavonoid luteolin regulated FOXO3. To our knowledge, this is the first study to demonstrate FOXO3 as a direct transcriptional activator of TREM2, thereby expanding the known regulatory network beyond previously reported regulators such as p53 and Nrf2 [66,67].

This study does not exclude the possibility that the anti-inflammatory effects of safflower leaves may also arise from low-abundance flavonoids or other bioactive constituents, such as phenolic acids and saponins, which may also confer benefits in AD. Further studies are needed to identify the active components and to define their relative contributions. As for FOXO3, the phosphorylation and acetylation of FOXO3 can impact its protein level [68,69], and these factors offer valuable directions for future research on how luteolin regulates FOXO3, not limited to the cellular localization. Furthermore, the pathology of AD involves not only microglial inflammation but is also accompanied by an impairment in microglial phagocytic function—a key mechanism for Aβ clearance [70]. Therefore, investigating the effects of safflower leaves or luteolin on microglial phagocytosis represents a critical future direction to fully elucidate the mechanism underlying their amelioration of cognitive deficits in APP/PS1 mice.

## 5. Conclusions

Our study reveals that safflower leaves ameliorate AD pathology by suppressing microglial inflammatory responses through a novel FOXO3-TREM2 axis, with its predominant flavonoid luteolin playing a central role. Our findings identify FOXO3 as a previously unrecognized regulator of *Trem2* transcription and broaden understanding of how the natural antioxidants safflower leaves ameliorate AD by modulating microglial inflammatory responses, thereby bridging traditional dietary interventions and molecular neuroprotection in AD. This work provides a mechanistic rationale for developing luteolin-based strategies to mitigate microglial dysfunction, and advances knowledge of *Trem2* transcriptional regulation.

## Data Availability

The original contributions presented in this study are included in the article and Supplementary Materials. Further inquiries can be directed to the corresponding authors.

## Supplementary Materials

The following supporting information can be downloaded at: https://www.mdpi.com/article/10.3390/antiox14121495/s1, Figure S1: Determination of optimal LPS treatment conditions for inducing inflammatory responses in RAW264.7 cells; Figure S2: 3D model video of Iba1 positive microglia; Table S1: Primers for real-time PCR; Table S2: siRNA sequence for knocking down; Table S3: Primers for site-directed mutagenesis PCR; Table S4: JASPAR database prediction of FOXO3 binding sites with the *Trem2* promoter; Supplementary raw data: western blot raw data.

## Abbreviations

The following abbreviations are used in this manuscript:

AD	Alzheimer’s disease
TREM2	triggering receptor expressed on myeloid cells 2
LPS	lipopolysaccharides
FOXO3	forkhead box protein O3
TNF-α	tumor necrosis factor-α
IL-1β	interleukin-1β
NO	nitric oxide
CD33	cluster of differentiation 33
ABCA7	ATP-binding cassette sub-family A member 7
SHIP1	Src homology 2 domain-containing inositol phosphatase 1
APOE	apolipoprotein E
CNS	central nervous system
DPPH	1,1-diphenyl-2-picrylhydrazyl
SLP	safflower leaves powder
WT	wild-type
PFA	paraformaldehyde
FBS	fetal bovine serum
H-DMEM	high glucose-Dulbecco’s modified eagle medium
MTT	3-(4,5-Dimethylthiazol-2-yl)-2,5-diphenyltetrazolium bromide
DMSO	dimethyl sulfoxide
IF	immunofluorescence
PBS	phosphate-buffered saline
BSA	bovine serum albumin
DAPI	4′,6-diamidino-2-phenylindole
CD68	cluster of differentiation 68
mIHC	multiplex immunohistochemistry
Iba1	ionized calcium-binding adaptor molecule 1
PMSF	phenylmethylsulfonyl fluoride
SYK	spleen tyrosine kinase
TLR4	toll-like receptor 4
ERK	extracellular signal-regulated kinase
TBST	Tris-buffered saline with Tween 20
SEM	standard error of the mean
ANOVA	analysis of variance
IL-6	interleukin-6
IC_50_	half maximal inhibitory concentration
CI	combination index
NOS2	nitric oxide synthase 2
COX-2	cyclooxygenase-2
Aβ	amyloid-β
JNK	c-Jun N-terminal kinase
