# Supplementary material for: Luteolin in Safflower Leaves Suppresses Microglial Inflammation Through FOXO3-Mediated Trem2 Transcription
Source: Antioxidants (Basel). 2025 Dec 12;14(12):1495. doi: 10.3390/antiox14121495 (PMC12729663; doi:10.3390/antiox14121495)
Supplement: Supplementary file 1 [file antioxidants-14-01495-s001.zip › Supplementary figure-20251124.pptx]

## Slide 1
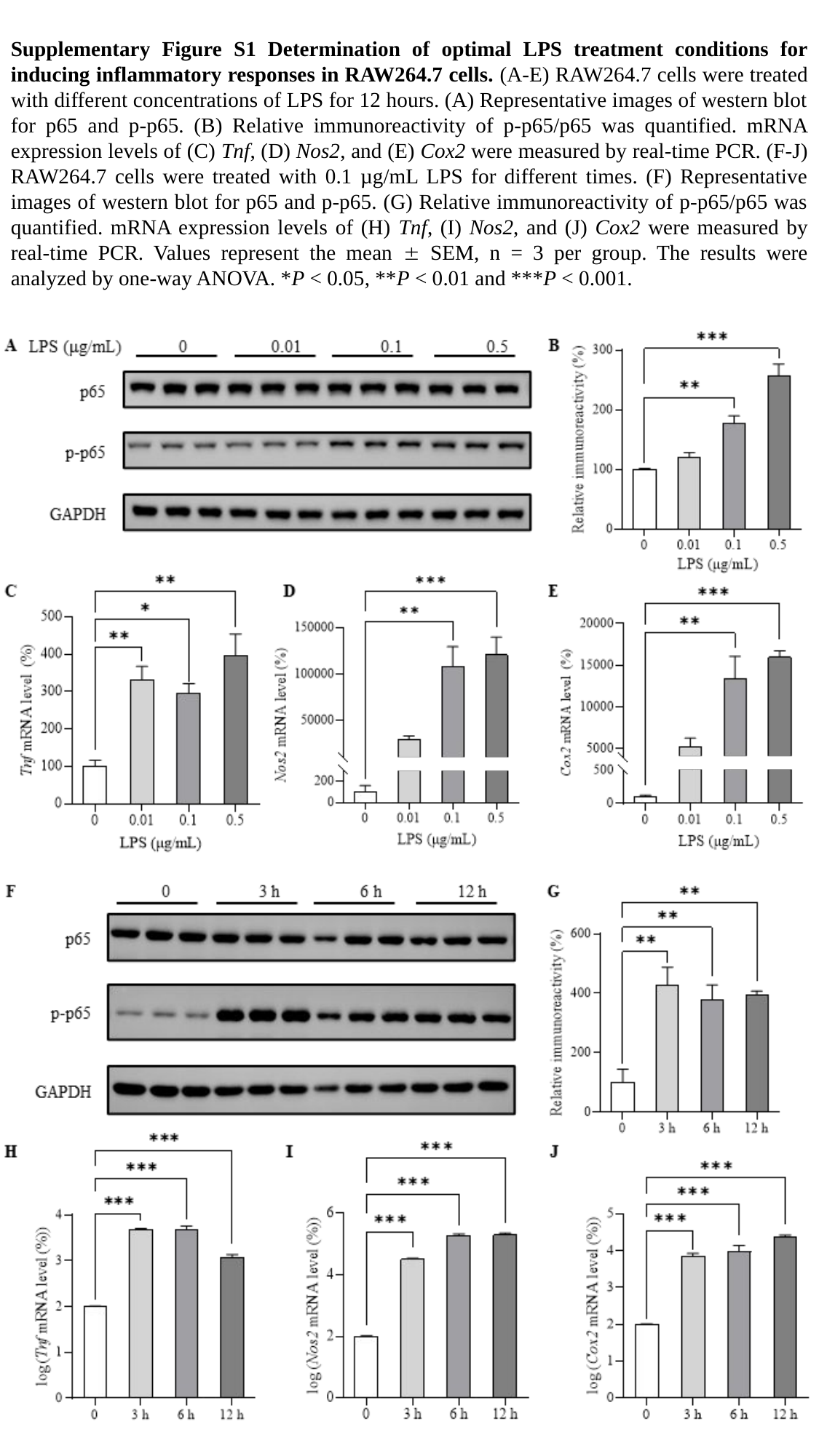

Supplementary Figure S1 Determination of optimal LPS treatment conditions for inducing inflammatory responses in RAW264.7 cells. (A-E) RAW264.7 cells were treated with different concentrations of LPS for 12 hours. (A) Representative images of western blot for p65 and p-p65. (B) Relative immunoreactivity of p-p65/p65 was quantified. mRNA expression levels of (C) Tnf, (D) Nos2, and (E) Cox2 were measured by real-time PCR. (F-J) RAW264.7 cells were treated with 0.1 µg/mL LPS for different times. (F) Representative images of western blot for p65 and p-p65. (G) Relative immunoreactivity of p-p65/p65 was quantified. mRNA expression levels of (H) Tnf, (I) Nos2, and (J) Cox2 were measured by real-time PCR. Values represent the mean ± SEM, n = 3 per group. The results were analyzed by one-way ANOVA. *P < 0.05, **P < 0.01 and ***P < 0.001.

## Slide 2
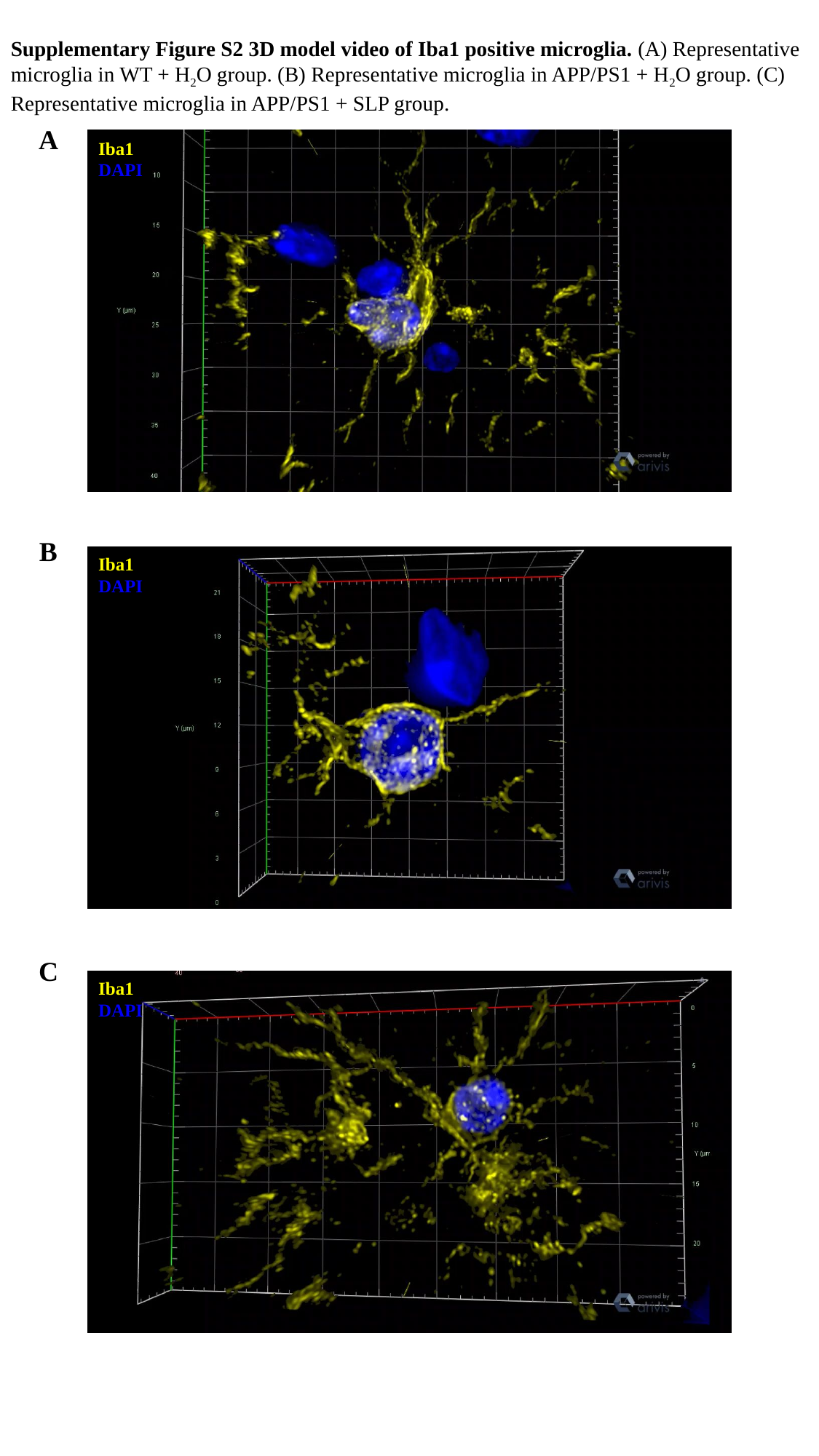

Supplementary Figure S2 3D model video of Iba1 positive microglia. (A) Representative microglia in WT + H2O group. (B) Representative microglia in APP/PS1 + H2O group. (C) Representative microglia in APP/PS1 + SLP group.
A
Iba1
DAPI
B
Iba1
DAPI
C
Iba1
DAPI
