# Supplementary material for: Luteolin in Safflower Leaves Suppresses Microglial Inflammation Through FOXO3-Mediated Trem2 Transcription
Source: Antioxidants (Basel). 2025 Dec 12;14(12):1495. doi: 10.3390/antiox14121495 (PMC12729663; doi:10.3390/antiox14121495)
Supplement: Supplementary file 1 [file antioxidants-14-01495-s001.zip › Supplementary raw data-20251124.pptx]

## Slide 1
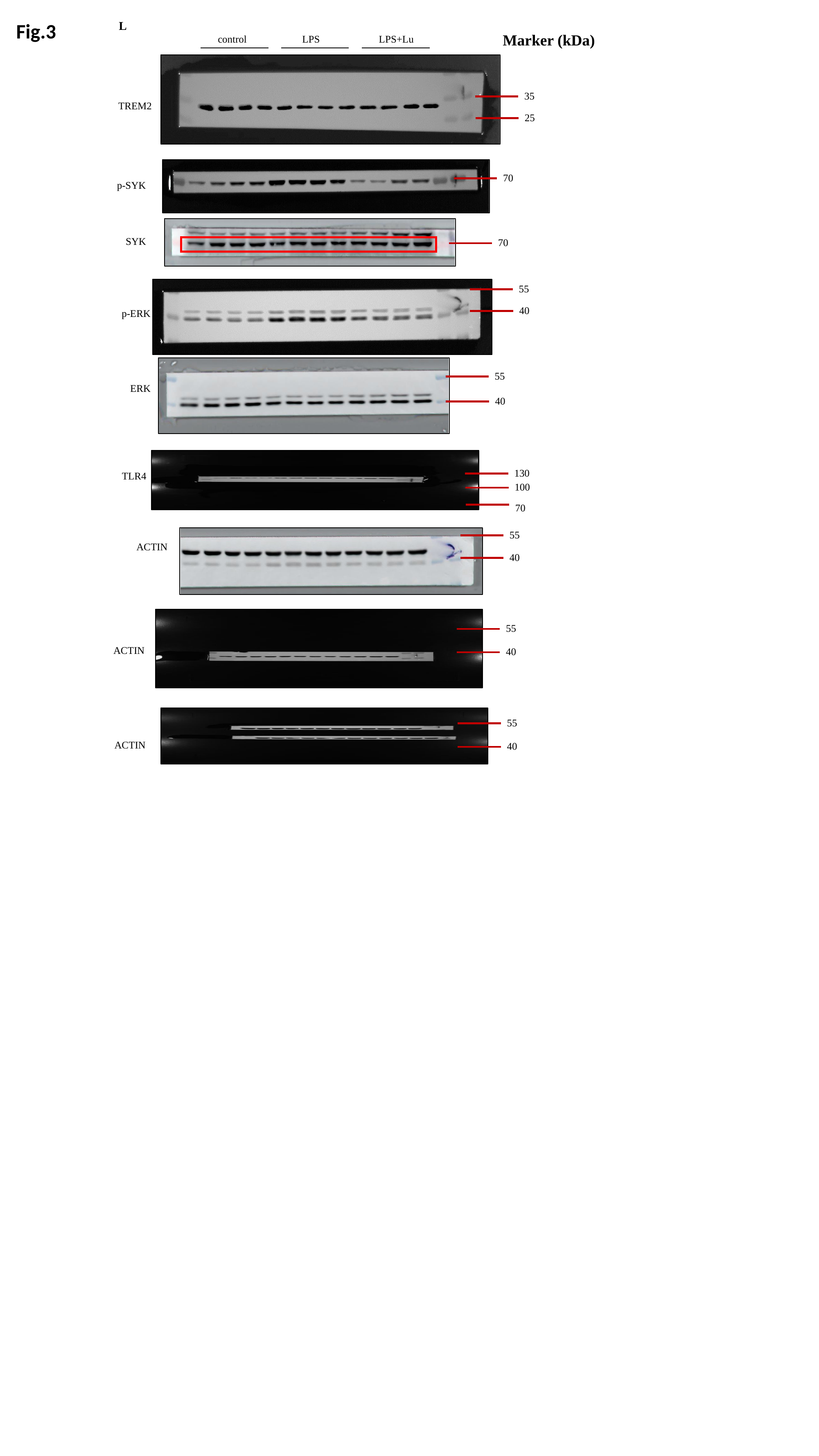

Fig.3
L
Marker (kDa)
 control	 LPS	 LPS+Lu
35
TREM2
25
70
p-SYK
SYK
70
55
40
p-ERK
55
ERK
40
130
TLR4
100
70
55
ACTIN
40
55
ACTIN
40
55
ACTIN
40

## Slide 2
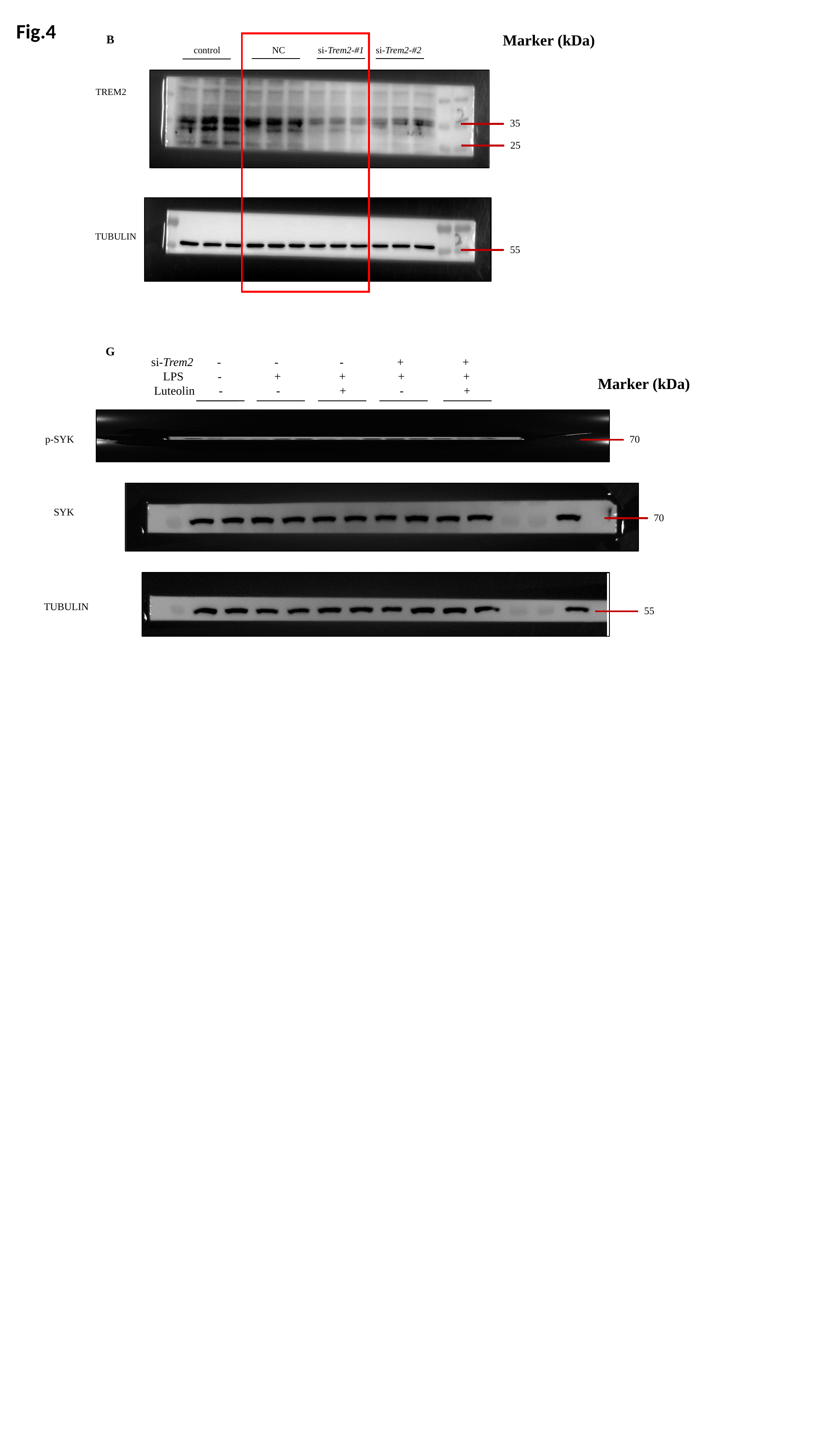

Fig.4
Marker (kDa)
B
 control NC si-Trem2-#1 si-Trem2-#2
TREM2
35
25
TUBULIN
55
G
si-Trem2 - - - + +
 LPS - + + + +
 Luteolin - - + - +
Marker (kDa)
p-SYK
70
SYK
70
TUBULIN
55

## Slide 3
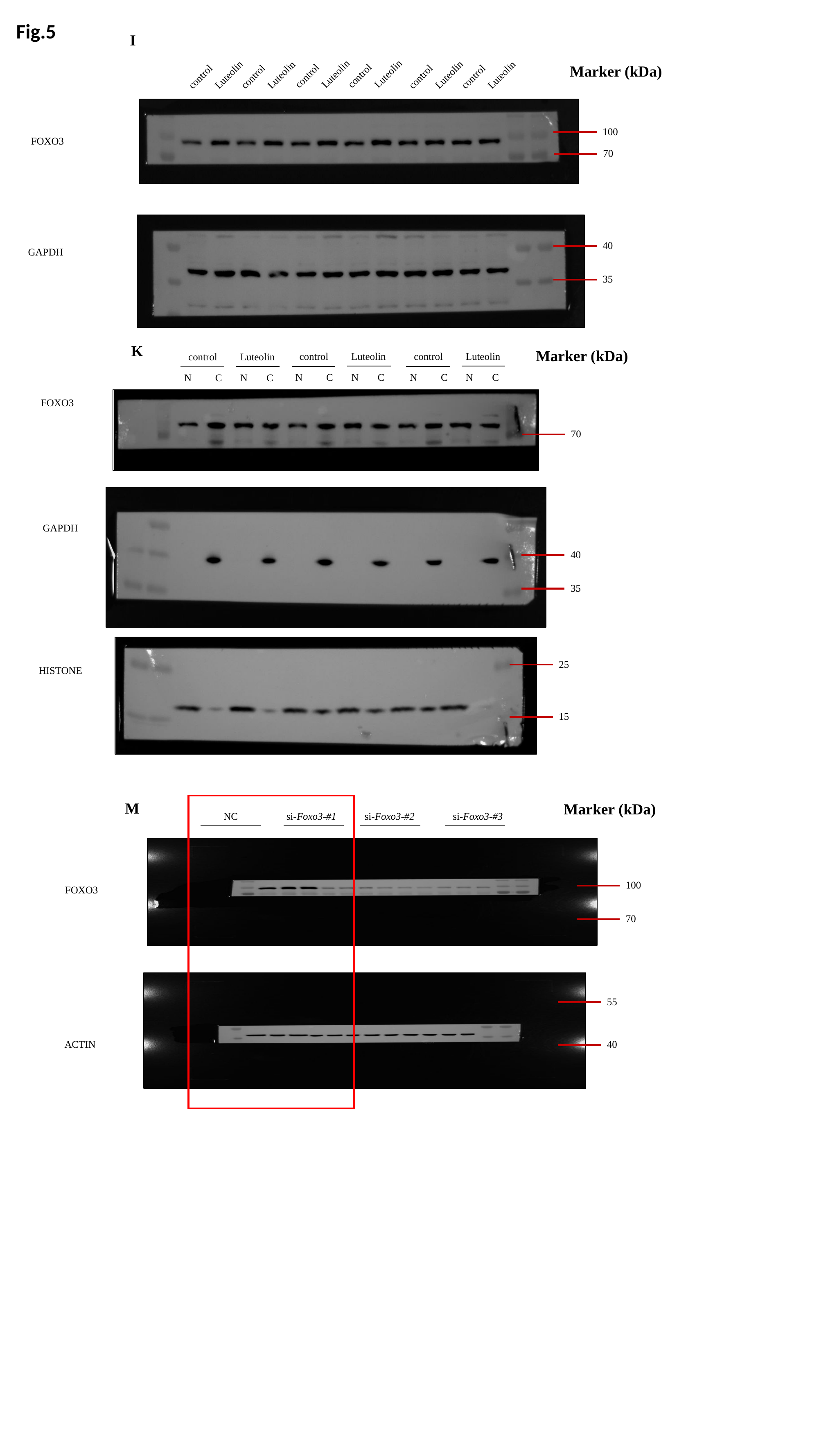

Fig.5
I
Marker (kDa)
control
Luteolin
control
Luteolin
control
Luteolin
control
Luteolin
control
Luteolin
control
Luteolin
100
FOXO3
70
40
GAPDH
35
K
Marker (kDa)
control
Luteolin
control
Luteolin
control
Luteolin
N
C
N
C
N
C
N
C
N
C
N
C
FOXO3
70
GAPDH
40
35
25
HISTONE
15
M
Marker (kDa)
NC si-Foxo3-#1 si-Foxo3-#2 si-Foxo3-#3
100
FOXO3
70
55
ACTIN
40
