# Supplementary material for: Luteolin in Safflower Leaves Suppresses Microglial Inflammation Through FOXO3-Mediated Trem2 Transcription
Source: Antioxidants (Basel). 2025 Dec 12;14(12):1495. doi: 10.3390/antiox14121495 (PMC12729663; doi:10.3390/antiox14121495)
Supplement: Supplementary file 1 [file antioxidants-14-01495-s001.zip › Supplementary table.pdf]

Supplementary Table S1 Primers for real-time PCR

Name	Forward Sequence	Reverse Sequence
Actb	GGCTGTATTCCCCTCCATCG	CCAGTTGGTAACAATGCCATGT
Tnfα	CCCTCACACTCAGATCATCTTCT	GCTACGACGTGGGCTACAG
Il6	TAGTCCTTCCTACCCCAATTTCC	TTGGTCCTTAGCCACTCCTTC
Il1β	GCAACTGTTCCTGAACTCAACT	ATCTTTTGGGGTCCGTCAACT
Cox2	GAGTGGTAGCCAGCAAAGCC	TTTAATTGGGAACCCTTCTTTGTT
Trem2	CTGGAACCGTCACCATCACTC	CGAAACTCGATGACTCCTCGG
Foxo3	CTGGGGGAACCTGTCCTATG	TCATTCTGAACGCGCATGAAG

Supplementary Table S2 siRNA sequence for knocking down

Name	Sense Sequence	Antisense Sequence
**si-*Foxo3***	GCACCAUGAAUCUGAAUGATT	UCAUUCAGAUUCAUGGUGCTT
**si-*Trem2***	GAACCGUCACCAUCACUCUTT	AGAGUGAUGGUGACGGUUCTT

Supplementary Table S3 Primers for site-directed mutagenesis PCR

Site	Forward Sequence	Reverse Sequence
**Mut1**	TCTGGCACAGCTCTGCGCCCAGGC TGGGGATGC	GCATCCCCAGCCTGGGCGCAGAGC TGTGCCAGA
**Mut2**	ACCAGAGATTGGCTGCGCCCCTAT CCCCTGCTG	CAGCAGGGGATAGGGGCGCAGCC AATCTCTGGT

Supplementary Table S4 JASPAR database prediction of FOXO3 binding sites with the *Trem2* promoter

Transcription Factor	Score	Start	End	Strand	Predicted sequence
**FOXO3**	8.7891	422	428	-	GGAAACA
**FOXO3**	8.7891	1473	1479	-	GGAAACA
**FOXO3**	7.1237	1423	1429	-	GTAAAGA
**FOXO3**	7.1104	66	72	-	GTTAACA
**FOXO3**	7.1104	67	73	+	GTTAACA
